# Evaluation of Ferric and Ferrous Iron Therapies in Women with Iron Deficiency Anaemia

**DOI:** 10.1155/2014/297057

**Published:** 2014-06-11

**Authors:** Ilhami Berber, Halit Diri, Mehmet Ali Erkurt, Ismet Aydogdu, Emin Kaya, Irfan Kuku

**Affiliations:** ^1^Division of Hematology, Department of Hematology, Faculty of Medicine, Medical School, Inonu University, 44280 Malatya, Turkey; ^2^Department of Internal Medicine, Medical School, Inonu University, 44280 Malatya, Turkey

## Abstract

*Introduction*. Different ferric and ferrous iron preparations can be used as oral iron supplements. Our aim was to compare the effects of oral ferric and ferrous iron therapies in women with iron deficiency anaemia. * Methods*. The present study included 104 women diagnosed with iron deficiency anaemia after evaluation. In the evaluations performed to detect the aetiology underlying the iron deficiency anaemia, it was found and treated. After the detection of the iron deficiency anaemia aetiology and treatment of the underlying aetiology, the ferric group consisted of 30 patients treated with oral ferric protein succinylate tablets (2 × 40 mg elemental iron/day), and the second group consisted of 34 patients treated with oral ferrous glycine sulphate tablets (2 × 40 mg elemental iron/day) for three months. In all patients, the following laboratory evaluations were performed before beginning treatment and after treatment. * Results*. The mean haemoglobin and haematocrit increases were 0.95 g/dL and 2.62% in the ferric group, while they were 2.25 g/dL and 5.91% in the ferrous group, respectively. A significant difference was found between the groups regarding the increase in haemoglobin and haematocrit values (*P* < 0.05). * Conclusion*. Data are submitted on the good tolerability, higher efficacy, and lower cost of the ferrous preparation used in our study.

## 1. Introduction

Iron deficiency is defined as the state in which the body iron is lower than the amount that is sufficient to maintain normal haemoglobin production and normal functions of iron-containing enzymes. Iron deficiency anaemia (IDA) is an important public health concern worldwide, particularly in developing countries in which nutritional problems are more common. Since this is the most frequent reason for anaemia, it should be kept in mind in the differential diagnosis of patients with anaemia. The cause of IDA is the failure of iron intake from food to meet the iron requirements of the body, and it is most commonly seen in women. Menstrual bleeding, pregnancy, abortion, and curettage are the most commonly encountered etiological causes of iron deficiency in women [1–3].

The reduced form (ferrous) is required for iron absorption, and the effects of reduced substances, such as ascorbate or succinate, on the iron valance (reduction of ferric iron) improve iron absorption. Phytates in cereals, tannins in tea, polyphenols in wine, antacids in milk, oxalate, and some antibiotics (tetracycline, e.g.) can form complexes with iron which do not resolve in water and can impede iron absorption [4, 5]. Achlorhydria, malabsorption states, and bypass through a gastrojejunostomy can cause iron deficiencies [6, 7].

To maintain the iron balance, 1.0–1.5 mg of iron absorption is required daily for men. However, an average of 60 mL/month of iron loss occurs in women during their menstrual cycles, and there is 0.4 mg of iron per millilitre of blood. Thus, women require an additional iron supplementation of approximately 30 mg per month. There is a tendency towards a decrease in iron storage during pregnancy, due to the increased maternal blood volume and the additional iron requirement for fetal haemoglobin synthesis. Therefore, the daily iron requirement increases to 5-6 mg/day during pregnancy. The most common cause of IDA is blood loss for both men and women, and the most frequent reasons for blood loss are gastrointestinal bleeding in men and menstrual bleeding in women. In menopausal women, the cause of IDA is the gastrointestinal system, unless proven otherwise. The gastrointestinal system should be evaluated in patients with IDA, even in the absence of a positive stool guaiac test or melena. IDA can be the first finding in right colon tumours or other occult cancers of the colon [8, 9].

For IDA treatment, the goals are to treat the underlying cause, correct the anaemia, and fill the iron stores. For this purpose, oral agents are generally preferred because of their ease of usage, low rate of adverse effects, and effectiveness. The routine approach in IDA management is to restore haemoglobin and haematocrit values by using full doses of oral agents over 3 months, followed by half doses over additional 3 months in order to replace the stored iron. Ferrous (Fe^+2^) and ferric (Fe^+3^) iron preparations are both used as oral agents [8–10]. However, Raja et al. and Jacobs reported that the absorption of Fe^+2^ iron from the intestine is 3 times higher than that of Fe^+3^ iron [4, 5].

Dose-dependent adverse effects, such as nausea, vomiting, abdominal pain, diarrhea, and constipation, can occur during the treatment of IDA by oral agents; however, these adverse events are rarely severe enough to discontinue the supplements. Symptomatic treatment, dose reduction, or ingestion after meals can generally relieve these adverse effects [10].

Iron therapy generally relieves fatigue and weakness within the first week, but reticulocytosis does not occur until 7–10 days after therapy begins. No elevation is seen in the haemoglobin level until 2 to 2.5 weeks after therapy, and a few months are needed to achieve normal haemoglobin values. Ferritin levels should be measured after the reconstruction of iron storages [11].

The aim of the present study was to evaluate Fe^+3^ and Fe^+2^ iron therapies in IDA treatment in women with regard to adverse events and efficiency. Using more effective and less expensive agents in IDA management will lead to rapid recovery and decreased costs.

## 2. Materials and Methods

The present study included 104 women who presented at the Haematology Outpatient Clinic of Inonu University School of Medicine and were diagnosed with iron deficiency anaemia after evaluation. The Fe^+3^ group (*n* = 54) received an oral Fe^+3^ protein succinylate flacon, while the second group (*n* = 50) received oral Fe^+2^ glycine sulphate tablets to be taken for 3 months.

In all patients, the following iron laboratory evaluations were performed before treatment began: complete blood count (CBC), serum iron level, total iron binding capacity (TIBC), transferrin saturation, serum ferritin level, and stool guaiac test, as well as parasite evaluations if necessary. Transferrin saturation was estimated by using the following formula: serum iron level/TIBC × 100.

In our study, the serum iron levels and TIBCs were measured via the colorimetric method by Olympus device (Germany), using an OSR6186 kit, and the serum ferritin levels were measured via the nephelometric method using the BNII device (Dade Behring, Germany). The CBCs were performed by using an LH750-ANA device (Beckman Coulter, USA). All analyses were performed on the same day by using blood samples drawn in the morning, after one night of fasting.

The following criteria were used to diagnose IDA in our female patients: haemoglobin < 12 g/dL, haematocrit < 35%, serum iron level < 50 *μ*g/dL, transferrin saturation < 10%, and serum ferritin level < 10 ng/dL. It was ensured that all criteria were met by our study patients.

The inclusion criteria were as follows: women aged 19–60 years who were diagnosed with IDA, the detection of the IDA aetiology and treatment of the underlying aetiology before iron therapy, absence of pregnancy, lack of comorbid disease (chronic disease anaemia, thalassemia, other haematological diseases, chronic renal failure, hypothyroidism, Addison's disease, malignancy, alcoholism, or gastrointestinal disease with impaired iron absorption), no acute or chronic infection, and no therapy with an iron preparation or blood product within 6 months prior to the investigation.

Before treatment, all patients were informed about the general principles of the study and the potential adverse effects of the iron preparations. All patients gave written informed consent. Because the diagnosis and treatment of the underlying aetiology are important for the success of treatment in iron deficiency anaemia, anamnesis (history, comorbid disease, hypermenorrhea, internal haemorrhoid, gastrointestinal bleeding, and nutritional characteristics), physical examination, and laboratory evaluations (stool guaiac test and parasite evaluations) were performed in all patients. Endoscopy and colonoscopy were performed in all postmenopausal women, even in the presence of a negative stool guaiac test, and therapies directed toward the underlying aetiology were completed before iron therapy. Since the cause of IDA was hypermenorrhea in most of the patients, hypermenorrhea therapy was arranged by the Obstetrics and Gynaecology Department of the Inonu University Medical School. The treatment of internal haemorrhoids was arranged according to the degree of the disease. Moreover, all patients were informed about the beverages and drugs which impair iron absorption.

The patients included were randomly assigned into 2 groups receiving either Fe^+2^ or Fe^+3^. Oral Fe^+3^ protein succinylate (40 mg elemental iron, twice daily) was initiated in Fe^+3^ group (*n* = 54), and Fe^+2^ glycine sulphate tablets (40 mg elemental iron, twice daily) were given to the second group (*n* = 50). It was recommended that the patients take the drugs before meals in each group for better absorption. The above-mentioned recommendations were in agreement with the pharmacological information of the drugs.

There were no adjuncts other than proteinaceous compounds, such as folic acid, ascorbic acid, and citric acid, in either preparation used. Protein succinylate and glycol sulphate, bound to the iron for better absorption, are two compounds with a similar protein structure. Additionally, there are 2 other preparations with identical elemental iron contents, which have adjuncts with similar protein structures but without adjuncts such as folic acid, ascorbic acid, or citric acid; however, they are not present in the drug market for comparison.

Patients were phoned and asked for using suitable form and dose of drug once a month. At the control visits (after 3 months), anamneses regarding the treatment period, adverse effects, additional drugs, and nutritional status were taken, and a physical examination was performed in all patients. Some patients attended a “control visit” before the 3-month control visit because of adverse effects, or for other reasons. At the end of the therapy, routine blood analyses (CBC, serum iron level, total iron binding capacity (TIBC), and serum ferritin level) were performed to compare the baseline values.

The exclusion criteria included missed control visits, noncompliance with the drugs due to any reason, development of comorbid disease during therapy, use of additional drugs or beverages which impair the absorption of the study drugs, treatment with erythrocyte suspension or iron preparation, development of severe bleeding or haemolysis, and detection of failure in the treatment of the underlying aetiology.

## 3. Statistics

In our study, statistical analyses were performed by using SPSS for Windows. Continuous variables were expressed as a mean ± standard deviation. Categorical variables were expressed as the number and percent. Normality for the continuous variables in the groups was determined using the Shapiro-Wilk test. The variables showed a normal distribution (*P* > 0.05); therefore, the paired and unpaired *t*-tests were used for intragroup and intergroup comparisons of the haematological parameters. The Pearson Chi square test was used to detect the aetiology underlying the IDA. Fisher's exact test was used to detect the groups regarding adverse drug effects, and *P* > 0.05 was considered to be statistically significant.

## 4. Results

Overall, 104 patients began this study, 54 patients in the first group receiving Fe^+3^ protein succinylate and 50 patients in the second group receiving Fe^+2^ glycine sulphate. Total 40 patients were excluded in agreement with various criteria (Table 1). Thus, 64 patients overall (30 patients in the Fe^+3^ group and 34 patients in the Fe^+2^ group) were included in the analyses.

In the Fe^+3^ group, the mean age was 40.7 ± 7.3 years. In the Fe^+2^ group, the mean age was 39.1 ± 6.4 years. No significant difference was found between the groups with regard to age (*P* > 0.05). In the evaluations performed to detect the aetiology underlying the IDA, it was found that, of the 30 patients, 20 had hypermenorrhea (66%), 6 had malnutrition (20%), and 4 had internal haemorrhoids (14%) in the Fe^+3^ group. In the Fe^+2^ group, it was found that, of the 34 patients, 23 had hypermenorrhea (67%), 7 had malnutrition (20%), and 4 had internal haemorrhoids (13%). No significant differences were found between the two groups regarding aetiology (*P* > 0.05).

In the Fe^+3^ group and Fe^+2^ group, haemoglobin (Hg), haematocrit (Htc), red blue cell (RBC), mean corpuscular volume (MCV), mean corpuscular haemoglobin (MCH), mean corpuscular haemoglobin concentration (MCHC), iron (Fe), TIBC, transferrin saturation, and ferritin levels were shown in Tables 2(a) and 2(b).

In the analysis which was the primary argument of our study, we compared the pre- and posttreatment laboratory values between the two groups in Table 3. Hg, Htc, and TIBC showed a significant increase in the Fe^+2^ group when compared to that in the Fe^+3^ group (*P* < 0.05). There were differences between the two groups regarding the increase in Ferritin, RBC, MCV, MCH, and MCHC values, and it was found that the Fe^+2^ group had a better response regarding the increase in MCV, MCH, and MCHC values but no significant differences (*P* > 0.05). No differences were found between groups regarding the other parameters.

The study groups were compared regarding adverse effects during therapy. Two patients refused to continue therapy due to epigastric pain in the Fe^+3^ group, while 2 patients in the Fe^+2^ group discontinued therapy due to epigastric pain (*n* = 1) and constipation (*n* = 1). These patients were excluded from the study. Two patients reported constipation after the initiation of the drug in the Fe^+3^ group. It was recommended to continue therapy and add a laxative agent in two patients who presented with constipation during therapy. In the Fe^+2^ group, 2 of the 34 patients reported adverse effects, including epigastric pain in one and constipation in the other, and symptomatic treatment was prescribed to these patients. Patients reporting adverse effects during therapy were cited that their symptoms were relieved by symptomatic therapy without causing noncompliance to the iron treatment. In conclusion, drug-related adverse effects developed in 4 (7.4%) of the 54 patients receiving Fe^+3^ and 4 (8.0%) of the 50 patients receiving Fe^+2^. No significant differences were found between the groups regarding adverse effects (*P* > 0.05).

## 5. Discussion

The therapeutic value of oral iron preparations is determined by the intestinal bioavailability and gastrointestinal tolerability of the iron content [12]; therefore, many studies have been conducted regarding the absorption and bioavailability of oral iron preparations. Widely accepted opinion suggests that the absorption of Fe^+2^ iron from the intestine is 3-fold higher than that of Fe^+3^ iron [4, 5]. Thus, World Health Organization recommends Fe^+2^ iron in the treatment of IDA [9, 13]. There are conflicting results in the studies comparing oral Fe^+2^ and Fe^+3^ preparations regarding the rate of success in the restoration of anaemia [14, 15]. In 1992, Glassman compared oral Fe^+3^ and Fe^+2^ iron preparations with distinct combinations (Fe^+2^ fumarate and polysaccharide-iron complex) regarding changes in haematological parameters, but the authors found no significant differences [16].

In 1993, Jacobs et al. found no significant differences regarding the increase in haemoglobin and haematocrit values between groups receiving 60 mg (daily) Fe^+2^ sulphate and 100 mg (twice daily) Fe^+3^ polymaltose complex. Less improvement was seen in anaemia in the group receiving the 100 mg (daily) Fe^+3^ polymaltose complex when compared to the other groups [17]. In 1994, Nielsen et al. compared Fe^+2^ sulphate and the ferric-polymaltose complex and found that there was no significant change in the mean haemoglobin value in the group receiving the ferric-polymaltose. However, a significantly increased mean haemoglobin value was detected in the group receiving Fe^+2^ sulphate over 4 weeks [18].

In a study in 1996, Casparis et al. compared 4 groups, including pregnant and postpartum women. The first group received 75 mg (twice daily, orally) of liquid Fe^+2^ gluconate, and the second group received 80 mg (daily, orally) of solid Fe^+2^ gluconate. The third group received 105 mg (daily, orally) of solid Fe^+2^ sulphate, and the fourth group received 80 mg (twice daily, orally) of liquid Fe^+3^ protein succinylate. After 30 days of therapy, no significant differences were observed among the groups regarding an increase in RBC, haemoglobin, haematocrit, and serum iron values [19].

In a study (in 2004) from Taiwan, in which Fe^+2^ fumarate and Fe^+3^ polysaccharide preparations were compared, Saha et al. found that Fe^+2^ fumarate was more effective after 12 weeks of therapy regarding improvements in the haematological parameters [20]. In a study from India, Saha et al. assigned 100 pregnant women into 2 groups to receive 120 mg of Fe^+2^ sulphate and 100 mg of Fe^+3^ polymaltose complex. They recommended the Fe^+3^ polymaltose complex for pregnant women, although there was no significant difference between the groups regarding improvements in haematological parameters after 8 weeks of therapy [21].

Ruiz-Argüelles et al., in a study from Mexico, reported that iron hydroxide polymaltose therapy failed in the treatment of IDA [22]. Aycicek et al. reported a new study to compare the total oxidant and antioxidant effects of different oral iron preparations in children with IDA. A total of 65 children with IDA were randomized to receive 5 mg Fe/kg/day of iron (II) sulphate (Fe^+2^ group, *n* = 33) or iron (III-) hydroxide polymaltose complex (Fe^+3^ group, *n* = 32). Healthy controls (*n* = 28) were also included in this study. The study concluded that Fe^+2^ sulphate (Fe^+2^) had a faster effect than Fe^+3^ polymaltose (Fe^+3^) on increasing the oxidant status in children with IDA [23].

In the treatment of IDA, gastrointestinal tolerability and the incidence of adverse side events are as important as bioavailability and efficiency when comparing the drugs used. There are several studies in the literature regarding gastrointestinal intolerance to oral iron preparations. Harvey et al. compared oral Fe^+3^ and Fe^+2^ iron preparations and found no significant differences between the groups regarding adverse effects [19]. In the study by Reddy et al., liquid Fe^+2^ gluconate was considered to be the safest supplement with regard to adverse effects [24]. Kavaklı et al. reported that both drugs were safe with regard to adverse effects and well tolerated, although the rate of gastrointestinal adverse effects was slightly higher in the group receiving Fe^+2^ fumarate. The authors noted that the inability to compare pure Fe^+2^ and Fe^+3^ iron preparations which did not include adjuncts, such as ascorbic acid, folic acid, or polysaccharide compounds, was an important limitation [20].

Saha et al. found that gastrointestinal adverse events were more common with Fe^+2^ sulphate therapy [21]. In a study in 2001, Harvey et al. suggested that gastrointestinal adverse effects were more frequently observed with Fe^+2^ iron when compared to Fe^+3^ iron, which could result from the production of more hydroxyl free radicals in the gastrointestinal mucosa. The authors recruited 23 patients (15 patients with inflammatory colon disease) with an intolerance to Fe^+2^ iron preparations and gave the patients Fe^+3^ trimaltol iron therapy. No adverse effects were detected in any of the patients, and significant increases were achieved in the haemoglobin and haematocrit levels with 3 months of therapy [24]. In another study, Kavaklı et al. evaluated the development of gastrointestinal adverse effects with the Fe^+3^ polymaltose complex and Fe^+2^ fumarate treatments in 100 women and reported that the Fe^+3^ polymaltose caused less adverse effects [25]. In 2004, Kavaklı et al. evaluated the development of oxidation-related toxicity and adverse effects in 2 groups of patients receiving either Fe^+3^ or Fe^+2^ iron. The authors found no significant differences between the groups [26].

When studies comparing oral Fe^+3^ and Fe^+2^ iron preparations were evaluated, with regard to the development of gastrointestinal intolerance, no definitive conclusion could be made about the superiority of any preparations. It was seen that Fe^+3^ iron preparations in the same form (solid-liquid) did not cause more adverse effects than Fe^+2^ iron preparations; however, it was also seen that they caused less intolerance in some studies.

In our study, 40 mg (twice daily; 0.5 hours before meals) oral Fe^+3^ protein succinylate flacons and 40 mg (twice daily; 0.5 hours before meals) oral Fe^+2^ glycine sulphate tablets were used; however, no significant difference was found regarding the adverse effects, and both preparations were found to be safe.

In evaluations regarding anaemia, haemoglobin and haematocrit are more valuable than RBC, MCV, MCH, and MCHC. Thus, we valued the increases in the haemoglobin and haematocrit levels, rather than those of the RBC, MCV, MCH, MCHC, serum iron, TIBC, transferrin saturation, and ferritin levels after 3 months of therapy, when compared to the baseline levels.

In our study, Fe^+2^ iron preparations were found to be superior to oral Fe^+3^ protein succinylate and Fe^+2^ glycol sulphate containing the same amounts of elemental iron. However, there are several oral Fe^+3^ and Fe^+2^ iron preparations with various forms. Given the different forms of preparations in the literature, it is difficult to make suggestions, such as “all Fe^+2^ iron preparations lead to better improvements in anaemia when compared to all Fe^+3^ iron preparations,” based on the comparison of the preparations used in our study.

The limitations of our study included a relatively small sample size (64 patients overall) at the end of a 6-month study period and an assessment of the patients only at the end of month 3. Larger and more comprehensive studies are required with more frequent controls (e.g., at months 0, 1, 3, and 6), which could include a greater number of patients and compare more preparations.

One interesting finding of our study was regarding cost. According to the 2013 year prices, the Fe^+3^ iron preparation was found to be more expensive than the Fe^+2^ iron preparation when the costs of 3 months of therapy were compared.

## 6. Conclusion

In conclusion, it was found that the Fe^+2^ and Fe^+3^ preparations used in our study were safe with regard to gastrointestinal intolerance; however, the Fe^+2^ was more effective and less expensive. Using more effective and less expensive agents in IDA management leads to a rapid recovery with decreased costs. Larger, multicentre studies should be performed on the absorption, adverse effects, and efficiency of oral iron preparations by evaluating scientific concerns before cost.

## Figures and Tables

**Table 1:** Total 40 patients were excluded in agreement with above-mentioned criteria.

Adverse effect	Fe^+3^ group	Fe^+2^ group
Epigastric pain	2	1
Constipation	0	1
Hypermenorrhea	9	6
Erythrocyte suspension	2	2
Not attending control visit	11	6
Total number of patients	24	16

**Table:** (a)

Parameters	Before treatment	After treatment	*P*
Hg (g/dL)	11.2	12.4	S**
Htc (%)	34.2	36.8	S
RBC (×10^12^/L)	4.3	4.6	S
MCV (fL)	79.8	82.4	S
MCH (pg/cell)	26.4	27.61	S
MCHC (g/dL)	33.3	33.3	NS*
Fe (*μ*g/dL)	18.3	68.2	S
TIBC (*μ*g/dL)	333	352.5	NS
Trans. sat. (%)	5.5	19.5	S
Ferritin (ng/dL)	9	12.3	S

*NS: not statistically significant; **S: statistically significant.

**Table:** (b)

Parameters	Before treatment	After treatment	*P*
Hg (g/dL)	10.3	12.6	S
Htc (%)	32.07	37.9	S
RBC (×10^12^/L)	4.37	4.70	S
MCV (fL)	72.5	82.1	S
MCH (pg/cell)	23	27	S
MCHC (g/dL)	31.5	33.2	S
Fe (*μ*g/dL)	22.7	61.9	S
TIBC (*μ*g/dL)	366.4	329.6	S
Trans. sat. (%)	6.2	18.52	S
Ferritin (ng/dL)	8.2	12.2	S

**Table 3:** Pre- and posttreatment laboratory values between the groups.

Parameter	Fe^+3^ group	Fe^+2^ group	*P*
Hg (g/dL)	0.95 ± 0.74	2.25 ± 0.94	S
Htc (%)	2.62 ± 2.07	5.9 ± 2.3	S
RBC (×10^12^/L)	0.24 ± 0.14	0.32 ± 0.33	NS
Ferritin (ng/dL)	4.13 ± 7.5	4.05 ± 10.2	NS
TIBC	19.5 ± 53.3	36.8 ± 71.9	S

## Abbreviations

IDA: Iron deficiency anaemia
Hg: Haemoglobin
Htc: Haematocrit
TIBC: Total iron binding capacity
Fe^+3^: Ferric
Fe^+2^: Ferrous
RBC: Red blue cell
Fe: Iron
MCV: Mean corpuscular volume
MCH: Mean corpuscular haemoglobin
MCHC: Mean corpuscular haemoglobin concentration.
